# When Right Goes Left: Phantom Touch Induced by Mirror Box Procedure in Healthy Individuals

**DOI:** 10.3389/fnhum.2021.734235

**Published:** 2021-12-01

**Authors:** Raffaella Ricci, Michela Caldano, Ilaria Sabatelli, Emanuele Cirillo, Roberto Gammeri, Ezgi Cesim, Adriana Salatino, Anna Berti

**Affiliations:** ^1^Department of Psychology, University of Turin, Turin, Italy; ^2^Department of Neuroscience, Dokuz Eylul University, Alsancak, Turkey; ^3^Institute of Neuroscience (IoN), Université Catholique de Louvain Brussels, Brussels, Belgium

**Keywords:** tactile awareness, multisensory integration, mirror image, bilateral touch representation, body ownership and disownership

## Abstract

In the present article, we investigated the possibility of inducing phantom tactile sensations in healthy individuals similar to those that we observed in patients after stroke. On the basis of previous research, we assumed that manipulating visual feedbacks may guide and influence, under certain conditions, the phenomenal experience of touch. To this aim, we used the Tactile Quadrant Stimulation (TQS) test in which subjects, in the crucial condition, must indicate whether and where they perceive a double tactile stimulation applied simultaneously in different quadrants of the two hands (asymmetrical Double Simultaneous Stimulation trial, Asym-DSS). The task was performed with the left-hand out of sight and the right-hand reflected in a mirror so that the right-hand reflected in the mirror looks like the own left-hand. We found that in the Asym-DSS trial, the vision of the right-hand reflected in the mirror and stimulated by a tactile stimulus elicited on the left-hand the sensation of having been touched in the same quadrant as the right-hand. In other words, we found in healthy subjects the same phantom touch effect that we previously found in patients. We interpreted these results as modulation of tactile representation by bottom-up (multisensory integration of stimuli coming from the right real and the right reflected hand) and possibly top-down (body ownership distortion) processing triggered by our experimental setup, unveiling bilateral representation of touch.

## Introduction

Tactile processing is a fundamental aspect of body ownership construction. It is characterized by both operations whose product remains implicit (i.e., linked to processes that do not reach the subject’s consciousness, e.g., Berti et al., 1999) and operations whose product becomes explicit and reported by the subject as conscious experience. In different domains, both in healthy participants and in brain damaged patients it has been shown that explicit (phenomenal) experience, although based on specific neural signal (Blakemore et al., 2000), can be nonetheless non-veridical. In other words, people can report experiences that are not related to actual events. For instance, in the motor domain, it has been shown that subjects can become aware of the movements they programmed and not of the action they actually performed, with vision deceiving proprioception (e.g., Fourneret and Jeannerod, 1998). Consistently with this observation, motor awareness can be reported *before* the actual execution of an action (e.g., Libet et al., 1983) and even in *absence* of any action (as in anosognosia for hemiplegia, see Pia et al., 2004; Berti et al., 2005; Berti and Pia, 2006; Garbarini et al., 2012). Also in the sensory domain, the non-veridical tactile experience can be observed and is particularly striking in brain-injured patients. Halligan and colleagues (Halligan et al., 1996, 1997) described a stroke patient who reported feeling touch when he watched a stimulus being applied to his affected limb. Abnormal sensation has also been observed in patients with pathological embodiment (a disturbance of the feeling of body ownership, Garbarini et al., 2020) who report to perceiving the tactile stimuli applied to someone else’s hand (positioned in egocentric perspective) they believe to be their own (Garbarini et al., 2014; Pia et al., 2020). Moreover, in the neurological literature, a phenomenon is described, called “synchiria”, where patients report to be touched on both hands when they are actually touched only on the ipsilesional hand (Medina and Rapp, 2008; Medina and Coslett, 2016). Another instance of unusual tactile experience is “allochiria”, whereby patients report a stimulus delivered on the contralesional hand to be experienced on the ipsilesional hand (Oberstainer, 1881; Kawamura et al., 1987; Young and Benson, 1992). More recently, we reported a new phenomenon we called “synchiric extinction” (Ricci et al., 2019). We used the Tactile Quadrant Stimulation test (TQS), where stimuli could be delivered to one of four quadrants previously identified on the participants’ hands, either to one (Single Stimulation trial, SS) or to both hands (Double Simultaneous Stimulation trial, DSS). Most importantly, during DSS, stimuli were delivered to asymmetrical positions. Patients had to verbally report their tactile experience and also had to point to the stimulated quadrants. Results showed that in DSS trials, at least 50% of the patients, although “correctly” reporting a bilateral tactile experience, erroneously pointed, on the contralesional hand, to the quadrant corresponding to the one stimulated on the intact hand. We interpreted these findings as a manifestation of pathological neuroplastic mechanisms, triggered by the brain lesion, unmasking bilateral touch representation following unilateral stimulation (Noachtar et al., 1997; Hansson and Brismar, 1999; Tamè et al., 2012, 2016) that would be inhibited in the healthy brain (Medina and Coslett, 2016). In stroke patients, hyperactivation of the healthy hemisphere (Kinsbourne, 1977; Johansen-Berg et al., 2002; Corbetta et al., 2005; Grefkes et al., 2008; Salatino et al., 2014; Gammeri et al., 2020) would abnormally activate, via inter-hemispheric transfer (Iwamura et al., 1994; Iwamura, 2000; Fabri et al., 2001; Eickhoff et al., 2008; van der Knaap and van der Ham, 2011; Ricci et al., 2012; Bagattini et al., 2015) homologous representations of the healthy side in the damaged hemisphere after ipsilesional tactile stimulation, thus producing contralesional phantom sensations. We also proposed that the relative weight of homotopic representations, in the damaged hemisphere, might be enhanced by stimulation of the affected hand, as it occurs in the phenomenon of stochastic resonance (SR), whereby adding noise to sub-threshold stimuli improves their detection (Collins et al., 1996; Perez et al., 2007, 2010). The above mechanisms would be responsible for synchiria, when abnormal activation of homotopic representations are supra-threshold, or synchiric extinction, with sub-threshold homotopic representations requiring to be enhanced by stimulation of the affected hand.

Thus synchiric extinction and synchiria support the evidence of bilateral touch representations (Tamè et al., 2012, 2016) and the idea that ipsilateral tactile representation would be sub-threshold (Ricci et al., 2019) and/or inhibited (Medina and Coslett, 2016) in the healthy brain.

A question we ask in the present article is whether it is possible to induce “phantom” sensation in normal subjects, similar to the one we described in patients, taking advantage of the well-known modulatory effect that vision can have over touch. We already know from previous experiments that vision not only improves many aspects of somatosensory processing when tactile stimulus is actually applied to participants’ body (e.g., Tipper et al., 1998, 2001; Pavani et al., 2000; Longo et al., 2011; Longo and Sadibolova, 2013; Tamè et al., 2013), but it can also induce the illusion of feeling touch on a fake hand, as in the Rubber Hand Illusion (Pyasik et al., 2019). Therefore the presence/absence of veridical/non-veridical tactile experience on the participants’ hands was assessed using an adapted version of TQS where we manipulated through the mirror box procedure the visuotactile stimulation applied on the participants’ hands. Subjects had to report tactile stimuli delivered to both hands in different quadrants while looking at the reflection of the right-hand into the mirror and having the left-hand out of sight.

We hypothesized that the feeling of touch on the right-hand together with the vision of touch on the same (right) hand into the mirror (where the right-hand looks like the left-hand) would bias the perception of the left-hand touch localization. Crucially, the expected left-hand errors would be of synchiric type (that is, the reported feeling of touch on the left-hand would be on the same quadrant of the one actually touched on the right-hand) and not simple mislocalization errors. We do not expect to find synchiric errors on the right-hand. Moreover, the comparison between putative synchiria during right-hand SS and synchiric extinction during asymmetrical DSS would inform us on whether the perception of phantom touch on the left-hand is exclusively driven by the vision of the right-hand in the mirror accompanied by tactile sensation of the same (right) hand, or whether touch of the left-hand is necessary to induce synchiric sensations. We expect to observe no differences between SS and DSS in the former case. On the other hand, we expect to observe phantom touch during DSS but not during right-hand SS if left-hand tactile stimulation is necessary to produce a phantom sensation in the same location stimulated on the right-hand. The influence of response modality on phantom sensations was also investigated.

## Methods

### Participants

Thirty healthy volunteers (mean ± SD, 29 ± 7; 19 women) participated in the study (Table 1). They had a normal or corrected-to-normal vision, and no history of neurological or psychiatric illness. Handedness was estimated using the Edinburgh Handedness Inventory (Oldfield, 1971) test, which ranges from −100% (completely left-handed) to + 100% (completely right-handed, see Table 1). Participants gave written informed consent to participate in the study, which was approved by the Ethical Committee of the University of Turin.

**Table 1:** Participants’ demographic and experimental data.

Participant	Sex	Age	Education (Years)	Edimburgh Test score	Order 1 = MC-P first
1	F	38	21	60%	1
2	M	22	17	71%	1
3	M	25	19	52%	1
4	F	20	16	100%	1
5	F	30	23	100%	1
6	M	24	18	100%	1
7	F	53	13	100%	1
8	M	33	16	100%	1
9	F	21	16	100%	1
10	F	23	16	83%	1
11	F	30	23	100%	1
12	F	30	23	100%	1
13	F	42	18	100%	1
14	F	21	16	100%	1
15	F	31	16	100%	1
16	M	27	16	75%	2
17	M	32	13	86%	2
18	M	24	18	100%	2
19	F	32	13	100%	2
20	F	20	16	75%	2
21	F	32	18	100%	2
22	F	35	26	100%	2
23	F	35	21	100%	2
24	F	34	19	100%	2
25	M	22	16	100%	2
26	F	31	24	100%	2
27	M	31	13	100%	2
28	M	32	13	100%	2
29	M	21	16	71%	2
30	M	34	15	100%	2

### Stimuli

**Figure 1:**
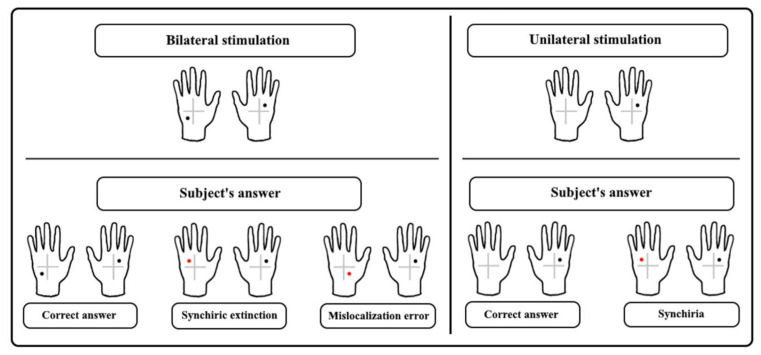
The upper panel shows examples of bilateral asymmetrical tactile stimulation and unilateral stimulation. In the lower panel response, examples are given showing the correct answer and three types of errors: synchiric extinction and mislocalization error regarding bilateral stimulation, and synchiria regarding unilateral stimulation.

The tactile stimuli were administered by the experimenter using calibrated nylon filament (Von Fray hair, size 15) to one of four quadrants, identified on the dorsum of each hand by a cross (5 × 5 cm) drawn on the center of the participant’s hand (Figure 1).

### Procedure

Participants sat with their hands on the table. Tape squares (1 × 1 cm) were placed on the table to mark the position where participants had to place the tip of the index finger for the right and the left-hand, 30 cm on either side of their sagittal midline.

Tactile stimuli were administered to one of the four quadrants on the left-hand (Single Stimulation Left-hand, SS-L), the right-hand (Single Stimulation Right-hand, SS-R) or both hands (Double Simultaneous Stimulation, DSS) to asymmetrical (Asym-DSS) or symmetrical (Sym-DSS) quadrants (Figure 1).

**Figure 2:**
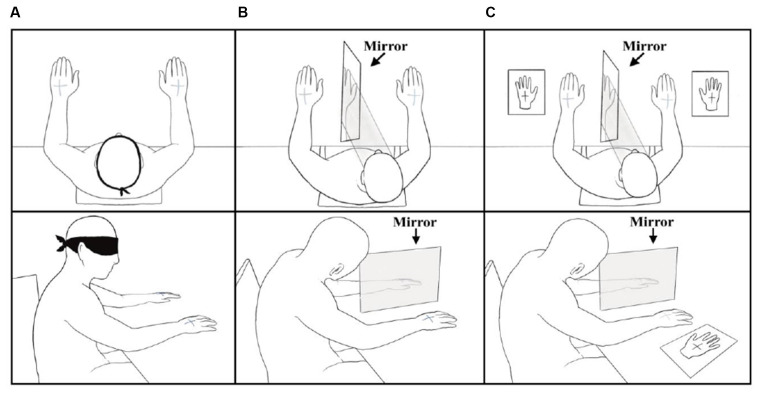
Top view and side view of the Baseline Condition **(A)**, Mirror Condition-Pointing **(B)**, and Mirror Condition-Silhouette** (C)**.

Stimuli were administered during three experimental conditions. In the *Baseline Condition* (BC) participants, blindfolded were asked to *verbally* report the side(s) of stimulation (left, right, or both) and then to *point* to the location(s) where they felt the tactile sensation(s), using the opposite hand (Ricci et al., 2019). During DSS trials participants used the right-hand first. After administration of the BC, participants underwent two *Mirror Conditions* (MC, see below), where a mirror (45 × 60 cm) was positioned perpendicularly to the subjects’ body, centered on their sagittal midline (Medina et al., 2018). In both MC, the subject’s hands were positioned at 30 cm of distance from the mirror, one to the right and one to the left of it (Figure 2). Participants were asked to look at the reflection of their right-hand in the mirror so that the mirror covered the left non-dominant hand. This experimental setting induced the perception that the right-hand mirror imagine fell exactly where the left-hand was positioned (Medina et al., 2018). In the* Mirror Condition-Pointing (MC-P*), after tactile stimulation, the subjects closed their eyes and *verbally* reported the side(s) (left, right, or both) of stimulation. Then they *pointed* to the location(s) where they felt the *tactile sensation(s)*, using the opposite hand. The *Mirror Condition-Silhouette (MC-S*) was identical to MC-P with the difference that participants reported the location(s) where they felt the sensation(s) using silhouettes of the right and the left-hand (14 × 8 cm) which were located on the table, 5 cm to the right and the left of the real hands (Figure 2C). Silhouettes were divided into four quadrants by a central cross (5 × 5 cm). For both MC, during DSS trials participants were not instructed on which hand to use first to report tactile stimuli. However, they tended to use the dominant hand first. Figure 2 depicts the three experimental conditions BC, MC-P, and MC-S.

The order of administration of MC-P and MC-S was counter-balanced across participants (Table 1). For each experimental condition stimuli were delivered according to two lists of 32 trials—i.e., eight trials (each quadrant was stimulated twice) for each stimulation condition—which follow a pseudo-random order. Participants underwent a total of 192 trials. The experiment lasted 60 min.

### Bodily Sensations Evaluation

To investigate participants’ subjective experience during mirror conditions, we audio-recorded spontaneous comments and observed the behavior of a subgroup of ten participants. In subjects not spontaneously verbalizing the experience, the experimenter asked one of the following questions: “what do you think?” or “how do you feel?” This session occurred before the first MC, and soon after participants started looking at the mirror right-hand reflection.

### Data Analyses

To assess the presence of synchiric extinction (i.e., errors due to localization of contralateral stimuli at homologous locations of ipsilateral stimuli) and synchiria (i.e., bilateral sensations during single stimulation) induced by the mirror, we analyzed separately stimulation conditions that could give rise to synchiric extinction and synchiria, i.e., Asym-DSS and SS trials, respectively. The analyses of Sym-DSS trials, which were not crucial for the aims of the study, are reported in the Supplementary Material. In the Asym-DSS, synchiric extinction was compared to mislocalization (i.e., stimulus localization in a location that was not touched in either hand) and classical extinction (i.e., failure to detect the left or the right stimulus), while in SS trials, synchiria was compared to mislocalization (i.e., stimulus localization in one of the quadrants not touched in the stimulated hand) and omissions. The number of errors constitutes the dependent variable (Ricci et al., 2019). See Figure 1 for a description of the types of errors.

Since data were non-normally distributed as assessed by the Shapiro-Wilk test, we used non-parametric Friedman and Wilcoxon tests (with Bonferroni correction when necessary) to compare within each *condition* (BC, MC-P, MC-S) the *type of errors* (Synchiric extinction, mislocalization, extinction/omissions) for each *hand* (left/covered hand vs. right/uncovered hand), and the two *hands* for each *type of* *error*. The analyses concerning the three main within-subjects factors (condition, error, and hand) and Spearman’s rho correlational analysis to assess the putative relationship between synchiric extinction for the left-hand and handedness are reported in the Supplementary Results.

## Results

### Asymmetrical DSS

**Figure 3:**
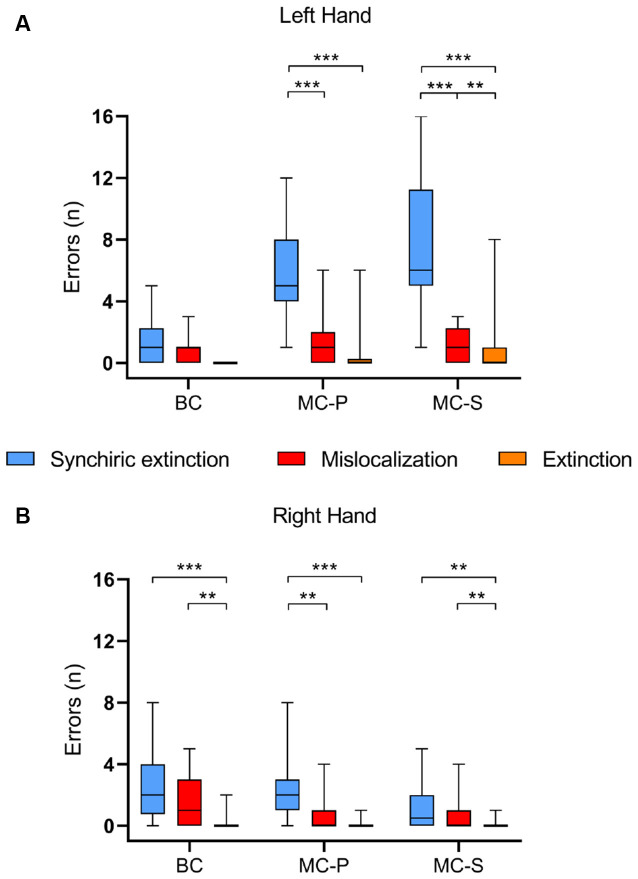
Group performances on the Baseline condition (BC), Mirror Condition-Pointing (MC-P), and Mirror Condition-Silhouette (MC-S). The graph depicts the median value of synchiric extinction, mislocalization errors, and extinction for each condition regarding **(A)** the left-hand and **(B)** the right-hand. Boxes represent the first to the third quartile, whiskers represent the data range. **p < 0.01; ***p < 0.0001.

Comparisons within each *condition* between *types of error* for each *hand* showed that, for the *left-hand*, in the *MC-S*, synchiric extinction was significantly greater than mislocalization [*z* = −4.630; *p* < 0.0001; *r* = 0.59] and extinction [*z* = −4.606; *p* < 0.0001; *r* = 0.59], and mislocalization was greater than extinction [*z* = −2.803; *p* < 0.01; *r* = 0.36]. Also, in the MC-P, synchiric extinction was greater than mislocalization [*z* = −4.417;* p* < 0.0001; *r* = 0.57] and extinction [*z* = −4.679; *p* < 0.0001; *r* = 0.60], without differences between these two last conditions after Bonferroni correction [*p* = 0.036 > 0.0167]. In the BC there were no differences between synchiric extinction and mislocalization and there was no extinction (Figure 3A).

For the *right-hand*, in MC-S, synchiric extinction did not differ from mislocalization [*z* = −1.784; *p* = 0.074; *r* = 0.23], while both synchiric extinction [*z* = −3.353; *p* < 0.01; *r* = 0.43] and mislocalization [*z* = −2.754; *p* < 0.01; *r* = 0.35] were greater than extinction. For MC-P, synchiric extinction was greater than mislocalization [*z =* −3.072; *p* < 0.01; *r* = 0.39] and extinction [*z* = −4.146; *p* < 0.0001; *r* = 0.53], without differences between these two last conditions after Bonferroni correction [*p* = 0.027 > 0.0167]. Finally, for BC, synchiric extinction and mislocalization did not differ between them, but both of them were greater than extinction (synchiric extinction: *z* = −4.218; *p* < 0.0001; *r* = 0.54; mislocalization: *z* = −3.398; *p* < 0.01; *r* = 0.43; Figure 3B).

Comparisons of each *type of* *error* between *hands* for each *condition*, revealed more synchiric extinction for the left-hand (behind the mirror) than for the right-hand in the two mirror conditions [MC-S: *z* = −4.685; *p* < 0.0001; *r* = 0.60; MC-P: *z* = −3.884; *p* < 0.0001; *r* = 0.50]. The two mirror conditions also showed more mislocalizations [MC-S: *z* = −2.840; *p* < 0.01; *r* = 0.37; MC-P: *z* = −2.130; *p* < 0.05; *r* = 0.27] and more extinction [MC-S: *z* = −2.588; *p* < 0.05; *r* = 0.33; *r* = 0.37; MC-P: *z* = −2.032; *p* < 0.05 *r* = 0.26] in the left than in the right-hand. Interestingly, an opposite result was found for synchiric extinction in BC, i.e., more bias in the right than in the left-hand [*z* = −2.238; *p* < 0.05; *r* = 0.28], and no differences for the other two types of bias.

To summarize, data showed induction of synchiric extinction by the mirror conditions in the left covered hand, and that within this hand, synchiric extinction was significantly greater than mislocalization. In addition, the type of mirror condition affected synchiric bias, with the silhouette condition producing a greater bias than the closed-eye pointing condition.

### Single Stimulation (SS)

In SS trials, in BC participants did not show any synchiria on the left-hand and a very small error (*M* = 0.03 *SD* = 0.18) on the right-hand. Also in the mirror conditions, synchiria was <0.3. Participants did not show any omission in BC and very small omission rate (<0.03) in the mirror conditions. They instead showed mislocalizations, with MC-S producing a greater bias than MC-P and BC. See Supplementary Results for details on this analysis.

### Bodily Sensations

As it emerged by a qualitative analysis of participants’ behavioral and verbal reactions (see Supplementary Results), participants expressed disorientation, astonishment, negative emotions, and, sometimes, some degree of amusement. They felt as if the mirrored image of the right-hand were the left-hand and that this feeling was quite uncomfortable. Thus these data revealed some sort of embodiment of the participants’ left-hand into the mirrored image of their right-hand. The participants’ verbalizations also convey a feeling of discomfort caused by the mirror experience.

## Discussion

In the present study, we evaluated if it was possible to induce phantom tactile sensations in healthy subjects similar to those observed in patients after stroke, based on the assumption that vision can, under certain circumstances, guide and influence tactile perception. To this aim, we used the TQS protocol in which subjects, in the crucial condition, must indicate whether and where they detected a double tactile stimulation applied simultaneously in different quadrants of the two hands. The task was performed with the left-hand out of sight (covered hand) and the right-hand (uncovered hand) reflected in a mirror placed so that the two hands were equidistant from the mirror. This situation induces the so-called mirror box illusion, whereby the right-hand reflected in the mirror looks like the own left-hand (Ramachandran et al., 1995).

Interestingly, we found that the vision of the right-hand reflected in the mirror and stimulated by a tactile stimulus, elicited on the left-hand, that received the stimulation in a different quadrant, the sensation of having been touched in the same position as the right-hand. In other words, we found in healthy subjects the same phantom touch effect that we previously observed in patients (Ricci et al., 2019). Here, we also observed enhanced effect in the silhouette condition, when the response mainly relied on vision.

The fact that vision can guide and even deceive tactile perception has been observed in the Rubber Hand Illusion (RHI, Botvinick and Cohen, 1998; Tsakiris and Haggard, 2005), where simultaneous stimulation of one’s own hand and of a corresponding rubber hand elicits the sensation that tactile stimuli are given on the rubber hand, with a consequent feeling of ownership over the rubber hand. In the RHI, the initial incongruence between touch, proprioception, and vision is resolved by reallocating the own hand on the position occupied by the rubber hand. Although some incongruence between touch, vision, and proprioception may also occur in our setup, the first important difference with respect to the RHI paradigm is that we do not apply continuous stimulation to induce an illusion. Our subjects are presented with one stimulus per trial. Although multisensory integration of conflicting stimuli, resulting in perceptual biases, does not necessarily require continuous stimulation (Ernst and Banks, 2002; Papeo et al., 2010; Takasugi et al., 2011; Liu and Medina, 2021), our paradigm also differs from the RHI because in the RHI, beyond the presence of a fake hand, completely unrelated to the body, the fake hand is of the same identity as the stimulated real hand (e.g., left rubber hand/left real hand). In addition, in the RHI only one real hand (hidden from vision) is stimulated.

More similar to our experimental situation is the protocol used by Petkova and Ehrsson (2009) where participants reported feeling touches on a right rubber hand when they saw it simultaneously stimulated with their left-hand. The authors explained their observation suggesting an automatic integration between visual, tactile, and proprioceptive information coming from the two hands which caused the transfer of sensation from the left-hand to the right rubber hand. This transfer would be mediated by neurons with bilateral tactile receptive fields in the parietal cortex (Iwamura et al., 1994, 2002; Iwamura, 2000). According to the authors, the tactile stimulation of the participant’s real hand may have activated ipsilateral somatosensory areas. When this *prolonged* activation was combined with the visual stimulation coming from the fake hand, the activation reached the threshold for conscious awareness for the stimuli applied to the fake hand. Likewise, in our experiment, stimulation and viewing of the right-hand in the mirror may have triggered a mechanism similar to that hypothesized by Petkova and Ehrsson. In our protocol, during asymmetric bilateral stimulation, a tactile localization bias might have arisen from automatic integration between contrasting information (felt touch on the left-hand and seen touch on the right-hand reflected in the mirror). This bias would be mediated by bilateral touch representations (Tamè et al., 2012, 2016; Schaefer et al., 2013). Specifically, right-hand stimulation would activate a sub-threshold ipsilateral somatosensory representation, that would reach the threshold for awareness (with transfer or duplication of sensation to the left-hand) when subjects see the right reflected hand. However, our experiment has fundamental differences from that of Petkova and Ehrsson. The first is that, in our paradigm, in the critical condition, both hands were stimulated and subjects indicated the position of the tactile perception on the *real* left covered hand even though the location was not the real one, but the one corresponding to the location stimulated on the real right-hand. So what happens in our case is the transposition/duplication of a tactile experience from a real hand to another real hand and not from a real hand to a fake hand. This, however, could have happened with the mediation of the mirror image of the right-hand that looks like the left own hand. We do not have a direct assessment of how much the reflected hand is felt as the own left-hand. However, although preliminary, the participant’s comments suggest a sort of incorporation of the mirror image of the right-hand, as a left-hand, into their body representation. We will specifically investigate this aspect in future studies.

We may speculate that the conflicting multisensory integration induced by our setup together with a possible “incorporation” of the reflected right-hand as the own left-hand might have induced the “phantom touch” on the left real hand. It must be noted that we found phantom touch only in double stimulation trial. That is, when the participants looked at the reflected image of the right-hand being touched, without receiving any stimulation on the real left-hand, they did not report any phantom sensation. This indicates that a single stimulation of the right-hand is not sufficient to induce a non-veridical tactile experience on the left-hand. Similarly to what we observed in patients after stroke (Ricci et al., 2019), stimulation of the left-hand is needed to feel a tactile stimulus on the left-hand on the same quadrant of the right-hand. It is possible that in healthy subjects, sub-threshold ipsilateral somatosensory representations of the right-hand, reinforced by the reflected vision of the same hand, may need the stimulation of the left-hand to reach awareness, as it occurs in the phenomenon of stochastic resonance (SR), whereby adding noise to subthreshold stimuli allows their detection (Perez et al., 2010). The processing of this stimulation would be therefore modulated by the bottom-up (multisensory integration of stimuli coming from the right real and the right reflected hand) and possibly top-down (body ownership distortion) influences giving rise to the phantom sensation reported in bilateral trials.

We also found a modulation of the phenomenon by response factors. The use of vision (silhouettes) to localize sensations boosted the phenomenon. Moreover, strong right-handedness was associated with decreased synchiric extinction, likely arising from decreased interhemispheric interaction (Christman et al., 2009). Finally, in the baseline condition, a greater bias occurred in the right-hand, implying the possibility of inducing an even greater effect in correspondence of the inverse set-up. These findings, in line with previous evidence (Ricci and Chatterjee, 2004; Ricci et al., 2005), suggest the contribution of output stages of spatial processing to stimulus awareness and warrant further in-depth investigation to comprehend the role played by the response and decision-making aspects to non-veridical tactile sensations (Takasugi et al., 2011; Badde et al., 2019).

In conclusion, this is the first evidence of transposition/duplication of tactile sensation from one real own hand to the other real own hand in normal subjects, demonstrating that it is possible to induce “phantom” experience outside a paradigm where alien and/or fake hands are used. The behavioral protocol we have proposed, if coupled with psychophysiological and neuroimaging techniques can represent an effective tool to deepen our knowledge on the physiological and anatomical aspects of multisensory integration and on the mechanisms underlying uni- and bilateral representations of touch.

## Data Availability Statement

The raw data supporting the conclusions of this article will be made available by the authors, without undue reservation.

## Ethics Statement

The studies involving human participants were reviewed and approved by Ethics committee of the University of Turin. The patients/participants provided their written informed consent to participate in this study.

## Author Contributions

RR, MC, and AB coordinated the study. RR, MC, AB, and AS designed the study. IS, EC, and EzC performed the experiments. MC and RR supervised data analyses. IS, RR, MC, and AS analyzed the data. AB and RR wrote the manuscript. MC, EC, IS, EzC, and AS critically reviewed the manuscript. All authors contributed to the article and approved the submitted version.

## Conflict of Interest

The authors declare that the research was conducted in the absence of any commercial or financial relationships that could be construed as a potential conflict of interest.

## Publisher’s Note

All claims expressed in this article are solely those of the authors and do not necessarily represent those of their affiliated organizations, or those of the publisher, the editors and the reviewers. Any product that may be evaluated in this article, or claim that may be made by its manufacturer, is not guaranteed or endorsed by the publisher.
