# Supplementary material for: When Right Goes Left: Phantom Touch Induced by Mirror Box Procedure in Healthy Individuals
Source: Front Hum Neurosci. 2021 Dec 1;15:734235. doi: 10.3389/fnhum.2021.734235 (PMC8671635; doi:10.3389/fnhum.2021.734235)
Supplement: Supplementary file 1 [file Data_Sheet_1.pdf]

When right goes left: phantom touch induced by mirror box procedure in healthy individuals

Raffaella Ricci *[1,†], Michela Caldano [1,†], Ilaria Sabatelli[1], Emanuele Cirillo[1], Roberto Gammeri[1], Ezgi Cesim[1,2], Adriana Salatino [1,3], Anna Berti *[1]

1. Department of Psychology, University of Turin, Turin, Italy

2. Department of Neuroscience, Dokuz Eylul Universit,. Izmir, Turkey

3. Institute of Neuroscience (IoN), Université Catholique de Louvain Brussels, Belgium

† These authors have contributed equally to this work and share first authorship

**Corresponding Authors:*

Raffaella Ricci, Department of Psychology, University of Turin, Turin, Italy, Via Verdi 10, 10124 Turin, Italy. Email: raffaella.ricci@unito.it

Anna Berti, Department of Psychology, University of Turin, Turin, Italy, Via Verdi 10, 10124 Turin, Italy. Email: annamaria.berti@unito.it

Supplementary Results

Asymmetrical DSS

Since data were non-normally distributed as assessed by the Shapiro-Wilk test, we used non-parametric Friedman and Wilcoxon tests (with Bonferroni correction when necessary) to analyze the within-subjects factors *conditions* (BC, MC-P, MC-S), *type of error* (Synchiric extinction, mislocalization, extinction/omissions), and *hand* (left/covered hand versus right/uncovered hand).

For Asym-DSS trials, separate Friedman tests revealed significant effect for the factors condition [$\chi2$ (2)=35.539; $p < .0001$] and error [$\chi2(2)=48.800$; $p <.0001$]. Wilcoxon signed-rank test for the factor condition revealed that overall participants produced significantly more errors for MC-S [M= 1.88; SD= .69; z= -4.342; $p < .0001$; r= .56] and MC-P [M= 1.67; SD= .60; z= -4.035; r= .52] than BC (M= 1.04; SD= .47), whereas the difference between MC-P and MC-S did not survive Bonferroni correction [z= -2.278; p= .023 > .016; r= .29]. For the factor type of error, Wilcoxon signed-rank test showed that synchiric extinction (M= 3.37; SD= 1.35) was significantly [z= -4.763; $p < .0001$; r= .61] greater than mislocalization (M= .99; SD= .61) and [z= -4.763; $p < .0001$; r= .61] classical extinction (M= .23; SD= .51). Moreover, mislocalization was greater than extinction [z= -4.036; $p < .0001$; r= .52]. The factor hand showed that overall participants committed more errors [z= -4.743; $p < .0001$; r= .61] for the left (M= 2.13; SD= .74) than the right (M= .92; SD= .45) hand.

The analyses conducted within each condition, to compare type of errors for each hand and to compare the two hands for each error are reported in the main text.

Single Stimulation (SS)

For SS trials, in BC participants did not show any synchiria on the left hand (i.e., right hand stimulation) and a very small error (*M*= 0.03 SD=0.18) on the right hand (i.e. left hand stimulation). Also in the mirror conditions synchiria was < 0.3 (MC-P: left-hand *M*= 0.07 SD=0.37; right-hand *M* = 0.27 SD= 1.11; MC-S: left-hand *M*= 0.07 SD=0.25; right-hand *M* = 0.30 SD= 0.92). In addition, in BC participants did not show any omission and very small omission rate (< 0.03) in the mirror conditions (MC-P: left-hand *M*= 0.03 SD=0.18; right-hand *M*= 0.03 SD= 0.18; MC-S: left-hand *M*= 0.03 SD=0.18; right-hand *M*= 0.00 SD= 0.00).

During SS participants mainly manifested mislocalization. Wilcoxon signed rank test revealed that the rate of mislocalization was significantly greater in the MC-S [*M*= 2.76; *SD*= 2.32; *z*= -4.021; *p* < .0001; *r*= .51] and MC-P [*M*= 1.96; *SD*= 1.59 *z*= -3.406; *p* < .01; r= .44] compared to BC [*M*= .96; *SD*= .87], while there was no difference between MC-S and MC-P [*z*= -1.783; *p*= .075]. Within the BC, for the mislocalization error, the right hand showed more errors compared to the left hand [*z*= -2.696; *p* < .01; *r*= .35], while there was no difference between the number of errors of left and right hand in the MC-P. Regarding the MC-S, the left hand showed more mislocalization errors compared to the right hand [*z*= -2.439; *p* < .05; *r*= .31].

Symmetrical DSS

For Sym_DSS trials, Wilcoxon signed-rank test for the factor *error* revealed that overall participants produced significantly more mislocalizations compared to extinction errors [z= -4.706; p < .0001; r = .61]. Overall errors did not show a significant difference between the two *hands* [p = .974], however, the Wilcoxon test showed that in BC, the right hand manifested significantly more errors than the left hand [z= -3.459; p < .01; r= .45], and the same result was true for MC-P [z= -2.366; p < .05; r = .30], but not for MC-S [p = .256].

Edinburgh correlations with left hand synchiric extinction

Spearman's rho correlation coefficient was used to assess the relationship between synchiric extinction for the left hand in the three conditions (BC, MC-P, MC-S) and the Edinburgh handedness inventory scores. A significant negative correlation was found for the synchiric error in MC-P [$r_s = -.37$, $p < .05$ N= 30], but not for BC or MC-S.

Bodily sensations

All participants when initially put in the first mirror condition spontaneously showed an odd, emotionally negative experience. All of them repeatedly moved their hidden hand, to test discordances between the right hand mirror image appearing as a left hand and the real left hand. They were quite bewildered and uncomfortable with the mirror image visually appearing as their left hand.

This observation was confirmed by the audio-recording of spontaneous or induced verbalizations of the subgroup of ten participants that are reported below:

Subject #21: "Oh my God, that's bad. It's a bad feeling, it looks like my left arm". The subject seems to be struggling to accept the mirror, in fact, before stimuli administration, she continued to move her left hand as if she wanted to make sure that the one she was seeing in the mirror was not the left one.

Subject #22: "It's an awful feeling, it feels as if you're seeing your own arm but you're not". The subject at the beginning of task performance struggled to accept the presence of the mirror and the perceptual deception.

Subject #23: "It's a weird feeling, but it’s funny. I'm sure I may do wrong though, so I need to focus."

Subject #24: No verbal comments, but in the presence of the mirror she had a quite bewildered look. In the meantime, she was doing some tests to become aware (to proof to herself) that the projected arm was not the left.

Subject #25: "That's weird, wait I have to figure it out and get used to it."

Soggetto #26: Bewildered look and wide eyes: "it's very weird!"

Subject #27. At the beginning, he did not spontaneously express any verbal comment, but he had an extremely visible bewildered look. To the experimenter’s request to express his feelings he replied: "it is very weird/strange and it is also a bad feeling because it is a deception".

Subject #28. Wide-eyed and gaze fixed on the mirror, the subject moved her hand covered by the mirror to make sure the moving hand was not the one reflected. Then she said: "That's a bad feeling, I'm enjoying it though".

Subject #29: “Oh my God, it is so weird, that's really bad/ugly!”

Subject #30: The subject, looking bewildered but also amused, stated: "I can't believe it, it's a weird feeling".
